# Role of Combined [^68^Ga]Ga-DOTA-SST Analogues and [^18^F]FDG PET/CT in the Management of GEP-NENs: A Systematic Review

**DOI:** 10.3390/jcm8071032

**Published:** 2019-07-13

**Authors:** Luciano Carideo, Daniela Prosperi, Francesco Panzuto, Ludovica Magi, Maria Sole Pratesi, Maria Rinzivillo, Bruno Annibale, Alberto Signore

**Affiliations:** 1 Nuclear Medicine Unit, ENETS Center of Excellence, Sant’Andrea University Hospital, 00189 Rome, Italy; 2 Digestive Disease Unit, ENETS Center of Excellence, Sant’Andrea University Hospital, 00189 Rome, Italy; 3 Department of Medical-Surgical Sciences and Translational Medicine, Faculty of Medicine and Psychology, Sapienza University of Rome, 00189 Rome, Italy

**Keywords:** somatostatin scintigraphy, FDG, Gallium-68, NEN, GEP, NET

## Abstract

Gastro-entero-pancreatic neuroendocrine neoplasia (GEP-NENs) are rare tumors, but their frequency is increasing. Neuroendocrine tumors normally express somatostatin (SST) receptors (SSTR) on cell surface, especially G1 and G2 stage tumors, but they can show a dedifferentiation in their clinical history as they become more aggressive. Somatostatin receptor imaging has previously been performed with a gamma camera using [^111^In]In or [^99m^Tc]Tc-labelled compounds, while [^68^Ga]Ga-labelled compounds and PET/CT imaging has recently become the gold standard for the diagnosis and management of these tumors. Moreover, in the last few years ^18^F-fluorodeoxyglucose ([^18^F]FDG) PET/CT has emerged as an important tool to define tumor aggressiveness and give relevant prognostic information, particularly when coupled with [^68^Ga]Ga-labelled SST analogues PET/CT. This review focuses on the importance of combined imaging with [^68^Ga]Ga-labelled SST analogues and [^18^F]FDG for the management of GEP-NENs.

## 1. Introduction

Gastro-entero-pancreatic neuroendocrine neoplasia (GEP-NENs) or neuroendocrine tumors (NET) are rare and heterogeneous diseases, with increasing incidence and prevalence over the last decades [1]. Their prognosis is affected by a number of factors, including the primary tumor site, grading and staging [2,3,4].

The vast majority of these tumors express somatostatin (SST) receptors (SSTR) on tumor the cell surface, a feature that may be used for both diagnostic and therapeutic purposes. In fact, in addition to conventional cross-sectional imaging procedures, somatostatin receptors functional imaging tests (FITs) (i.e., ^68^Ga-labelled SST analogues, ^111^In-labelled SST analogues and ^99m^Tc-labelled SST analogues) are recommended in GEP-NEN patients at the time of disease diagnosis, as well as during patient follow-up [5].

Hybrid positron emission tomography and computed tomography with ^68^Ga-labelled SST analogues ([^68^Ga]Ga-DOTA-SST PET/CT) is considered the gold standard technique in GEP-NENs, due to its high sensitivity and specificity, which are reported to vary between 91–95% and 82–97%, respectively [6].

Positron emission tomography/computed tomography with ^18^F-fluorodeoxyglucose ([^18^F]FDG PET/CT) has been suggested as an alternative tool for tissue sampling for the assessment of the aggressiveness of tumors, and it has shown prognostic value in NENs [7].

In the present review, the role of [^68^Ga]Ga-DOTA-SST PET/CT and [^18^F]FDG PET/CT in NENs was investigated, focusing on the impact of their combined use in the clinical practice.

### 1.1. Conventional Somatostatin Receptors Imaging

Somatostatin receptors (SSTRs) are highly expressed on neuroendocrine cells. Octreotide was the first synthetic somatostatin analogue used in nuclear medicine to image SSTRs expression. For many years, SSTR imaging has been performed as octreotide scan with a gamma camera, although, in the last few years, PET/CT has been established as the best technique to image SSTRs.

Somatostatin receptor scintigraphy (SRS) with [^111^In]In-pentetreotide (Octreoscan^®^, Mallinckrodt Medical, St. Louis, MO, USA), first performed in 1989, is based on the specific binding of a radiolabeled somatostatin analogue (octreotide) to high affinity somatostatin receptors (mainly type 2) expressed by most neuroendocrine tumors. Imaging is generally performed at four and 24 h after the i.v. administration of Octreoscan^®^ with a two-headed gamma camera equipped with a medium energy collimator. SRS shows a good accuracy for whole body imaging and has been routinely used for the diagnosis and follow-up of neuroendocrine tumors [8,9].

The principal limitation of the use of SRS with Octreoscan^®^ is the low spatial resolution of the gamma camera that mainly affects the evaluation of small lesions, particularly in organs with a high physiological uptake (for example the liver). The introduction of SPECT/CT hybrid imaging has improved the accuracy of SRS—increasing its spatial resolution and the anatomic localization of pathologic sites of increased uptake due to CT co-registration, with a reduction of false positive results [10].

At the beginning of this century, two new ^99m^Tc-labelled SST analogues were introduced: [^99m^Tc]Tc-N4-[Tyr3] Octreotate (Demotate^®^, POLATOM, Otwock, Poland) and [^99m^Tc]Tc-EDDA/HYNIC-[Tyr3] Octreotide (Tektrotyd^®^, POLATOM, Otwock, Poland) [11], both of which are widely used in clinical practice with major advantages over [^111^In]In-Octreotide [12,13,14,15,16,17,18,19,20,21,22,23,24,25,26,27].

### 1.2. PET/CT with ^68^Ga-labelled Peptides

SRS-PET with ^68^Ga-labelled peptides has recently become the gold standard in the diagnosis and management of well differentiated neuroendocrine tumors. It has proven to be better than SRS imaging with gamma-emitting isotopes because of its higher sensitivity compared to Octreoscan. [28,29]

Furthermore, the use of PET/CT, as compared to SPECT/CT, offers higher spatial resolution, lower scan times and a lower patient radiation exposure per scan [30,31].

The radiopharmaceuticals developed for SRS-PET imaging consist of gallium-68 (a β+ emitting isotope with 68 min of half-life, obtained by a ^68^Ge/^68^Ga generator or by using low energy cyclotrons), a chelator—1,4,7,10-Tetraazacyclododecane-1,4,7,10-tetraacetic acid (DOTA) and a somatostatin analogue—NaI^3^-Octreotide (NOC), Phe^1^-Tyr^3^-Octreotide (TOC) or Tyr^3^-Octreotate (TATE). [^68^Ga]Ga-DOTA-NOC, [^68^Ga]Ga-DOTA-TOC, and [^68^Ga]Ga-DOTA-TATE, despite their different affinity for somatostatin receptors SSRs (they bind especially type 2, but also type 3 and type 5), have shown no differences in clinical practice; the three of them are widely used [32]. DOTA-TATE, DOTA-TOC and DOTA-NOC can also be labelled with Lutetium-177 (^177^Lu) and Yttrium-90 (^90^Y) for peptide receptor radionuclide therapy (PRRT), although DOTA-TATE is the most frequently used in clinical practice.

For SRS-PET, fasting is not required, and the scan starts generally 45–60 min after the intravenous administration of the labelled compound. Some authors suggest discontinuing “cold” octreotide therapy to avoid a possible SSTR blockade [32]. Physiological [^68^Ga]Ga-DOTA-SST analogue biodistribution includes the uncinate process of the pancreas, spleen, liver, adrenal glands, pituitary gland and urinary tracts. Possible pitfalls include inflammatory processes (leucocytes and macrophages express SSR2), splenosis, osteoblastic activity (degenerative bone disease, fracture, hemangioma, epiphyseal growth plates), and meningiomas. In addition, other tumors can overexpress SSTR, such as pheochromocytoma, paraganglioma and neuroblastoma [33]. Previous studies found that SUV_max_ values were significantly higher in patients with well-differentiated neuroendocrine carcinomas than with poorly differentiated carcinomas, whereas no correlation was found between SUV_max_ and Ki67 [34].

Overall, almost 90% of G1–G2 GEP-NENs present a positive finding due to the high SSTR expression on cell surface of these tumors. Recently, a negative correlation between tumor proliferative activity, expressed by Ki67, and [^68^Ga]Ga-DOTA-TATE, expressed as SUV_max_, has been observed in a retrospective analysis including 126 GEP-NENs [35]. The proposed SUV_max_ cut-off able to discriminate between G1–G2 vs G3 GEP-NENs was 11.2, thus suggesting that patients with a lower SUV_max_ value present a significant higher risk of having a more aggressive and highly proliferating G3 NEN.

A possible correlation between SUV_max_ and Ki67 has also been investigated by other studies, however, with heterogeneous findings. In the paper by Partelli and coworkers, the median SUV_max_ value varied between 31.5 and 53.5 in G1–G2 pancreatic NENs, whereas it was 16.5 in the G3 subgroup [36]. A prognostic role of SUV_max_ was proposed by Ambrosini et al., who observed a significantly longer progression-free survival in patients with an SUV_max_ value > 38 compared with those with a lower SUV_max_ value, in a series of G1–G2 pancreatic NENs (*p* = 0.002) [37]. Indeed, a lower cut-off value was proposed in the study by Sharma et al., in which the authors observed significantly different progression-free survival curves when patients were stratified according to SUV_max_ values with a cut-off of 14.5 [38].

All together, these results show that, although it is clear that a high SUV_max_ value means a better clinical outcome, the best cut-off value that is able to definitively stratify patients in different subgroups with different prognosis still needs to be identified.

Nevertheless, there is solid scientific evidence showing the positive impact of PET/CT with [^68^Ga]Ga-DOTA-SST analogues on GEP-NEN clinical management, particularly in detecting distant metastases [39]. A recent systematic review and metanalysis, including 14 studies, showed that PET/CT findings resulted in management change in 44% of patients, thus confirming the pivotal role of this technique in the management of NEN patients [30].

### 1.3. PET/CT Imaging with [^18^F]FDG

[^18^F]FDG (fluorodeoxyglucose) is the most common radiopharmaceutical used in PET imaging, especially for oncologic purposes. FDG is a glucose analogue that is actively transported by specific glucose transport proteins (GLUT, particularly GLUT1 and 3) into the cell and then phosphorylated by hexokinase. After phosphorylation, glucose normally enters the glycolysis pathway, but FDG cannot and remains trapped into the cell. Tumor cells, due to their higher metabolic activity, show an increased number of glucose transporters and hexokinase, leading to a higher FDG uptake than normal tissues [40]. PET images are generally acquired one hour after the intravenous injection of [^18^F]FDG (^18^F-half life: 109 min). Patients are required to fast at least six hours before injection, and blood glucose levels must not exceed 200 mg/dL, although 180 mg/dL is desirable. [^18^F]FDG PET/CT plays a very small role and has low sensitivity in small growing well differentiated neuroendocrine tumors (G1 and G2), but its role is emerging in the evaluation and management of high-grade NENs (G3). Indeed, with time, NENs may show a de-differentiation, losing their ability to express somatostatin receptors and increasing their metabolism and FDG avidity. Many studies have demonstrated a positive correlation between Ki67 expression and [^18^F]FDG SUV_max_ [41,42]. This finding suggests that [^18^F]FDG PET/CT has an important prognostic value in high grade NENs.

[^18^F]FDG PET/CT is commonly performed in aggressive NENs, as there is emerging evidence that the presence of increased glucose metabolism in NENs correlates with the aggressiveness of tumors and bad prognosis [7]. Though a clear relationship between [^18^F]FDG-PET/CT positivity and an unfavorable clinical outcome has been reported, particularly in aggressive G3 tumors, its role in more indolent G1 and G2 GEP-NENs still remains unclear. The European Neuroendocrine Tumors Society guidelines do not recommend [^18^F]FDG-PET/CT in NEN patients unless a G3 grading is present [43,44]. Conversely, several papers have reported its utility in patients with low grade (G1–G2) tumors—in whom it may provide useful prognostic information—in order to improve patients’ clinical management. [^18^F]FDG PET/CT sensitivity in G1–G2 GEP NENs is reported to range between 40% and 60%, whereas it increases to almost 95% in G3 tumors [42,45]. However, [^18^F]FDG PET/CT positivity in GEP-NEN lesions does not depend on grading alone—it also depends on tumor aggressiveness, differentiation, and GLUT expression [45].

According with European Neuroendocrine Society grading system [46,47], the grade 1 NENs group includes tumors with very low proliferative activity, their Ki67 limit being <3% or ≤2% in pancreatic or gastrointestinal primaries, respectively. Grade 2 (G2) NENs are diagnosed when tumors have a higher Ki67 value, though it still remains below 20%. A Ki67 value of > 20% leads to a diagnosis of G3 tumors (in the pancreatic primaries, G3 NENs are further classified according with tumor morphology in NEN G3 if well-differentiated or NEC G3 if poorly-differentiated) [48].

Patients’ prognosis dramatically depends on grading systems. In fact, G1 tumors are considered indolent diseases with an excellent long-term clinical outcome even in the setting of advanced disease. Their five-year survival rates are 60–80% and 40–60% in pancreatic and gastrointestinal primaries stage IV diseases, respectively. On the opposite side, G3 NENs, particularly if poorly-differentiated morphology is present, need to be considered aggressive diseases, with five-year survival rates ranging between 10% and 30% [2,4].

Based on these considerations, these tumors are expected to have a very different glycolytic activity, resulting in an extremely different probability to have [^18^F]FDG PET/CT positivity, which is common in G3 NENs, whereas it is infrequent in less proliferating tumors.

The availability of an accurate non-invasive diagnostic tool, such as [^18^F]FDG PET/CT, which is able to predict tumor behavior, may help in the early identification of those patients with unfavorable clinical outcome. This is even more significant considering that when disease progression is documented, a tumor grading increase may occur throughout the disease course in up to 25% of patients [49,50].

## 2. Combined [^68^Ga]Ga-DOTA-SST Analogues and [^18^F]FDG PET/CT in GEP-NENs

In the last few years, many authors have suggested to combining both [^18^F]FDG PET/CT and SRS-PET/CT for the management of neuroendocrine tumors, particularly G2 and G3. This combined analysis can provide useful information on tumor heterogeneity, the characterization of SSTRs expression, and tumor grade, thus guiding clinicians to the proper treatment options.

Our aim was to clarify, according to data available in the literature, if combined imaging should be recommended in the routine clinical practice for the management of GEP-NENs. For this review, we included only studies regarding GEP-NENs in which combined imaging, with both [^68^Ga]Ga-DOTA-SST analogues and [^18^F]FDG-PET/CT, was performed. All other papers about neuroendocrine neoplasms that did not focus on GEP-NENs were excluded. Only original articles were included, while other types of publications (reviews, case reports, and others) were excluded, such as papers in languages other than English.

**Figure 1:**
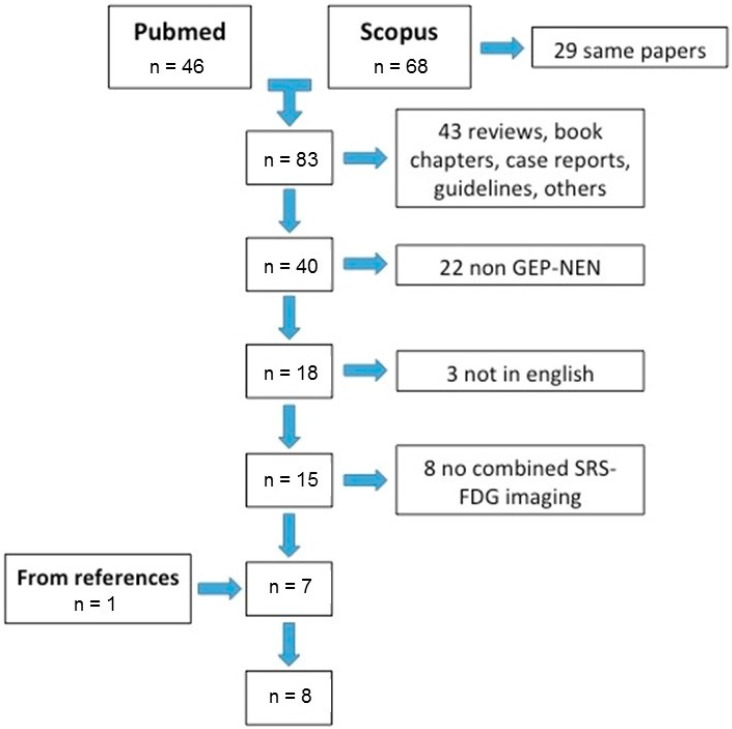
Flowchart of included papers.

Searching on PubMed and the Scopus database for papers that analyze the role of combined imaging in GEP-NEN using the following terms [FDG and 68GA and (NEN or NET or GEP)], we found 134 papers; 29 were duplicates, 43 were reviews, book chapters, editorials, comments, case reports, or other non-eligible types, 22 were not about GEP-NEN, three were not in English, and eight did not compare [^68^Ga]Ga-DOTA-SST analogues and [^18^F]FDG PET/CT. We overviewed the remaining seven works, plus one that we added after searching from the references of these papers, for a total of eight original articles (Figure 1).

Kayani et al. [42] evaluated the distribution of [^68^Ga]Ga-DOTA-TATE PET/CT in NETs and compared its performance with the [^18^F]FDG PET/CT in 38 patients affected by primary or recurrent NEN. According to the tumor histology, based on Ki67 and the mitotic index, all patients were classified into high, intermediate, or low grade tumors. Both imagings were performed within three weeks of each other, and histopathology was available in all patients. Tumors were classified according to the SUV_max_ value of both examination and the number of the lesions. It was found that the uptake of [^68^Ga]Ga-DOTA-TATE was greater than [^18^F]FDG in low grade NEN (SUV_mean_ 29 vs 2.9), while [^18^F]FDG uptake was higher than [^68^Ga]Ga-DOTA-TATE (SUV_mean_ 11.7 vs 4.4) in high grade NET. A significant correlation between predominant tumor uptake of [^68^Ga]Ga-DOTA-TATE or [^18^F]FDG and proliferation and tumor grade was also observed; tumor size did not significantly affect tracer uptake. The researchers concluded that well-differentiated NENs have grater avidity for [^68^Ga]Ga-DOTA-TATE, and poorly differentiated NENs have greater avidity for [^18^F]FDG.

Partelli et al. [36] analyzed the effect of combined [^68^Ga]Ga-DOTA-NOC PET/CT and [^18^F]FDG PET/CT on treatment management for patients with pancreatic NENs (pNENs), and they evaluated the correlation between the uptake of dual tracers and tumor grade. The combined PET/CT examination was performed on the same day. Patients were then divided in two groups: The first one included patients who had only a positive [^68^Ga]Ga-DOTA-NOC scan, while the other included patients positive at both [^68^Ga]Ga-DOTA-NOC and [^18^F]FDG PET/CT or only at the latter. The study demonstrated that, despite the fact that the use of combined PET/CT showed areas of different tumor grading among the same lesions, its routine use does not influence the choice of treatment strategy except in selected patients affected by pNEN with Ki67 > 10. Tumor grade, symptoms, and previous clinical history are among the factors that mainly influence the therapeutic strategy.

Naswa et al. [51] compared [^68^Ga]Ga-DOTA-NOC PET/CT with [^18^F]FDG PET/CT in patients with gastro-entero-pancreatic (GEP) NENs. They collected data from 51 patients and showed that [^68^Ga]Ga-DOTA-NOC has a higher diagnostic accuracy in finding primary and metastatic disease.

Abdulrezzak et al. [52] gave a contribution to the combined imaging approach with [^68^Ga]Ga-DOTA-TATE and [^18^F]FDG by measuring several volumetric parameters instead of SUV_max_ only, as there are many factors that can influence this parameter. These new parameters are the metabolic tumor volume (MTV) and the total lesion glycolysis (TLG) values in [^18^F]FDG PET/CT), the somatostatin receptor density (SRD) and the total lesion somatostatin receptor expression (TLSRE) values in [^68^Ga]Ga-DOTA-TATE PET/CT. In this study, authors included the primary tumor or metastatic lesions of 41 patients with NEN. The volumetric parameters were measured in addition to SUV_max_ and SUV_mean_ values. Patients were categorized into three groups on the basis of the Ki67 proliferation index of the tumor. They found that the [^68^Ga]Ga-DOTA-TATE SUV_max_ and TLSRE values of the primary tumor were higher than the [^18^F]FDG SUV_max_ and TLG values in the G1 NET group. In contrast, [^18^F]FDG SUV_max_ and TLG values of the primary tumor were higher than the [^68^Ga]Ga-DOTA-TATE SUV_max_ and TLSRE values in the G3 NET group.

Thapa and his group [53] performed combined imaging using either [^99m^Tc]Tc-Hynic-TOC or [^68^Ga]Ga-DOTA-TATE scans with [^18^F]FDG in 50 patients with metastatic GEP-NEN before and after PRRT therapy with [^177^Lu]Lu-DOTA-TATE. They found that FDG positivity, both in high and in low grade NEN, was associated with a worse response to radionuclide therapy and poor prognosis. They concluded that combined imaging can help to better identify patients that can benefit from PRRT.

Cingarlini et al. [54] studied a group of 35 patients with pancreatic well differentiated NET (G1–2) that underwent surgery for a localized or oligometastatic disease which was performed in the same day combined imaging with [^68^Ga]Ga-DOTA-TOC and [^18^F]FDG PET/CT. SRS-PET was positive in 33 of 35 patients, while 21 of 35 scans showed a FDG positivity whose percentage was higher in the G2 than in the G1 group. Moreover, the SUV_max_ values at FDG were greater in G2 than in G1 tumors, and FDG positive lesions tended to be larger than negative ones and showed a higher probability to have locoregional lymph nodes involvement and distant metastases. With these data, authors suggested that a pre-operative evaluation with combined imaging, in cases without consensus for surgical therapy, can distinguish patients who can benefit from other treatment options.

Recently, Chan et al. [55] proposed a new grading scheme for metastatic NEN by using both SRS-PET/CT and [^18^F]FDG PET/CT imaging based on a new score called the “NETPET Score”. This score allowed to identify five categories of patients: P1, with positive SRS-PET/CT only; P5, with positive [^18^F]FDG PET/CT only; and P2–P4, intermediate cases with both positive SRS-PET/CT and [^18^F]FDG PET/CT. P0 was for patients with both negative scans.

Authors found that the NETPET Score significantly correlates with tumor grade and overall survival, gives important prognostic information, and can identify patients that may benefit from PRRT. Based on these considerations, dual radiopharmaceutical PET imaging might be used as a prognostic biomarker. However, this needs to be validated in a larger prospective series of patients.

Zhang et al. [56] analyzed 83 patients with GEP-NEN divided in three groups based on the Ki67 index and mitotic count: Well-differentiated NENs group A (Ki67 < 10%), group B (Ki67 > 10%), and poorly differentiated NECs group C. Patients underwent both [^68^Ga]Ga-DOTA-TATE and [^18^F]FDG PET/CT. This retrospective study showed that while [^68^Ga]Ga-DOTA-TATE was useful for staging and follow-up, [^18^F]FDG showed a correlation with the aggressiveness of the tumor. [^68^Ga]Ga-DOTA-TATE uptake was also related to a good prognosis. By contrast, tumors with a worse prognosis were those solely with [^18^F]FDG uptake. Furthermore, they found that the sensitivity of the dual tracer (94%) was higher than that solely with [^68^Ga]Ga-DOTATATE and [^18^F]FDG. This finding was more evident in NET (Ki67 > 10%) than in NEC.

Taking all the reported data together, it appears clear that both techniques together play an important prognostic role in NENs, the clinical outcome being more favorable in patients with positive SRS-PET/CT and negative [^18^F]FDG PET/CT (Table 1 and Table 2).

**Figure 2:**
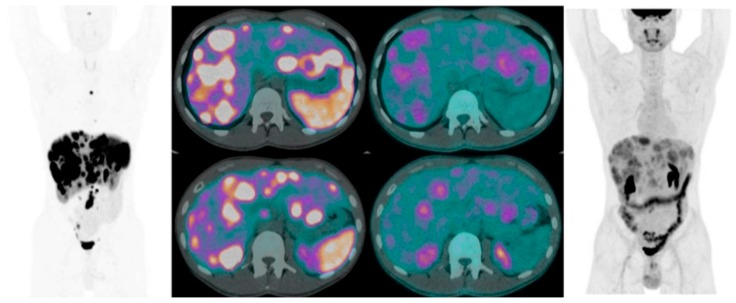
Twenty-four-year-old male patient with newly diagnosed ileal neuroendocrine neoplasia (NEN) with liver, lymph node and bone metastases. Combined imaging with [^68^Ga]Ga-DOTA- NaI^3^-Octreotide (NOC) PET/CT (left) and [^18^F]FDG PET/CT (right) show the heterogeneity of the disease.

**Figure 3:**
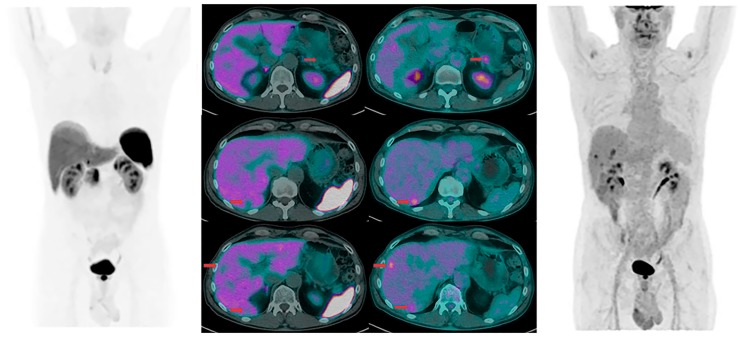
Sixty-seven-year-old male patient with newly diagnosed pancreatic neuroendocrine tumors (NET) with liver metastases. All lesions are positive at [^18^F]FDG PET/CT (right) and negative at [^68^Ga]Ga-DOTA-NOC PET/CT (left), indicating a more aggressive and un-differentiated disease.

Furthermore, dual PET/CT imaging may provide relevant information concerning disease heterogeneity which cannot be assessed by a single bioptic sampling in a given metastatic lesion [Figure 2 and Figure 3].

## 3. Conclusions

The combined use of SRS-PET/CT and [^18^F]FDG PET/CT still remains a challenge for physicians dealing with NENs. Assessing dual radiopharmaceutical PET/CT as a single parameter allows one to consider the two techniques as complementary rather than competitors, as previously suggested [57]. Using the dual tracer FITs, two different aspects of tumor biology may be explored: SSTR expression and glucose metabolism. SRS-PET/CT and [^18^F]FDG PET/CT may be used for precise staging of patients with metastatic tumors, in which metastatic lesions may present heterogeneous metabolic activity and somatostatin receptor expression [42].

Though, there is solid scientific evidence confirming the clinical role of these procedures in patient management, the optimal selection of patients who would benefit from their combined use still remains debated. Large prospective studies with homogeneous series of NEN patients are required to resolve this unmet need.

Nevertheless, based on major findings from this systematic review, it emerges that the combined use of SRS-PET/CT and [^18^F]FDG PET/CT should be considered in the following clinical scenarios:

(i) At the time of initial diagnosis: In those patients with intermediate tumor proliferative activity (i.e., G2 tumors); if there is a heterogeneous SSTR expression among different tumor lesions; and in non-functioning tumors when patients have tumor-related symptoms (i.e., pain and weight loss).
(ii) During follow-up: In addition to conventional radiological imaging at the time of first disease restaging after changing anti-proliferative medical treatment; at the time of disease progression after prolonged stable disease; and in case of a discrepancy between conventional radiological evaluation and clinical/biochemical assessment.

## Figures and Tables

**Table 1:** Summary of publications in which gastro-entero-pancreatic neuroendocrine neoplasia (GEP-NENs) are studied with ^68^Ga-labelled somatostatin (SST) analogues ([^68^Ga]Ga-DOTA-SST) analogues and ^18^F-fluorodeoxyglucose ([^18^F]FDG).

Title	Comments and Conclusion	Reference
Clinical and Prognostic Value of PET/CT Imaging with Combination of ^68^Ga-DOTA-TATE and [^18^F]FDG in Gastroenteropancreatic Neuroendocrine Neoplasms.	Clinical value of the dual PET/CT imaging in GEP/NEN	[56]
Dual Somatostatin Receptor/FDG PET/CT Imaging in Metastatic Neuroendocrine Tumors: Proposal for a Novel Grading Scheme with Prognostic Significance	New grading system for metastatic NET based on combined SSRI and FDG PET scans, with prognostic significance, that can change therapeutic decision.	[55]
Role of Combined ^68^Ga-DOTATOC and ^18^F-FDG Positron Emission Tomography/Computed Tomography in the Diagnostic Workup of Pancreas Neuroendocrine Tumors	Combined imaging has a role in pre-surgical evaluation of PanNENs	[54]
Performance of ^177^Lu-DOTATATE-Based Peptide Receptor Radionuclide Therapy in Metastatic Gastroenteropencreatic Neuroendocrine Tumor: A Multiparametric Response Evaluation Correlating with Primary Tumor Site, Tumor Proliferation Index, and Dual Tracer Imaging Characteristics	Combined imaging and FDG positivity predict PRRT response and give prognostic information	[53]
Combined Imaging with ^68^Ga-DOTA-TATE and [^18^F]FDG PET/CT on the Basis of Volumetric Parameters in Neuroendocrine Tumors	The role of the new volumetric parameters in NET	[52]
The Role of Combined ^68^Ga-DOTA-NOC and ^18^FDG PET/CT in the Management of Patients with Pancreatic Neuroendocrine Tumors	Tumor grade, symptoms and previous clinical history are the factors that mainly influence therapeutic strategy	[36]
Dual Tracer Functional Imaging of Gastroenteropancreatic Neuroendocrine Tumors Using ^68^Ga-DOTA-NOC PET-CT and [^18^F]FDG-PET-CT	Dual tracers can demonstrate the tumor burden independently on the level of differentiation	[51]
Functional Imaging Of Neuroendocrine Tumors With Combined ^68^Ga-DOTA-TATE (Dota-DPhe1,Tyr3-octreotate) and [^18^F]FDG PET/CT	The role of the 2 tracer may be complementary in mapping patients with metastatic tumors.	[42]

**Table 2:** Characteristics of selected publications.

Reference	Nr of Patients	Research Type	Grading	Imaging Techniques
[56]	83	Prospective	G1, G2, G3	[^68^Ga]Ga-DOTA-TATE and [^18^F]-FDG PET/CT within 2 weeks
[55]	62	Retrospective	G1, G2, G3	[^68^Ga]Ga-DOTA-TATE and [^18^F]-FDG PET/CT within 31 days
[54]	35	Retrospective	G1 and G2	[^68^Ga]Ga-DOTA-TOC and [^18^F]-FDG PET/CT in the same day
[53]	50	Retrospective	G1, G2, G3	[^99m^Tc]Tc-Hynic-TOC scintigraphy or [^68^Ga]Ga-DOTA-TATE and [^18^F]FDG PET/CT, distance not specified
[52]	41	Prospective	G1, G2, G3	[^68^Ga]Ga-DOTA-TATE and [^18^F]-FDG PET/CT within 1 month
[36]	49	Retrospective	G1, G2, G3	[^68^Ga]Ga-DOTA-NOC and [^18^F]-FDG PET/CT in the same day
[51]	51	Retrospective	Not specified	[^68^Ga]Ga-DOTA-NOC and [^18^F]-FDG PET/CT within 15 days
[42]	38	Retrospective	G1, G2, G3	[^68^Ga]Ga-DOTA-TATE and [^18^F]-FDG PET/CT within 3 weeks
